# P-1180. Effects of a Novel Bacterial Topoisomerase Inhibitor on Type II Topoisomerases in Bacillus anthracis

**DOI:** 10.1093/ofid/ofaf695.1373

**Published:** 2026-01-11

**Authors:** Chelsea Mann, Jason West, J Matthew Meinig, Mark Mitton-Fry, Neil Osheroff

**Affiliations:** Vanderbilt University School of Medicine, Nashville, TN; The Ohio State University, Columbus, Ohio; USAMRIID, Fort Detrick, Maryland; The Ohio State University, Columbus, Ohio; Vanderbilt University School of Medicine, Nashville, TN

## Abstract

**Background:**

The tier 1 agent, *Bacillus anthracis* (etiological cause of anthrax) is a highly transmissible pathogen that causes morbidity and mortality. The intentional release of anthrax spores from either wild-type or drug-resistant strains would constitute a severe threat to public and military health. Novel Bacterial Topoisomerase Inhibitors (NBTIs) are emerging antibacterials that target the type II topoisomerases, gyrase and topoisomerase IV. The NBTI pharmacophore promotes a binding mode that allows for the evasion of target-mediated fluoroquinolone (FQ) resistance. OSUAB0284 is a pre-clinical NBTI candidate that shows potent anti-staphylococcal activity in cultured cells and a neutropenic mouse model.^1,2^ Gepotidacin is an advanced NBTI-type first-in-class triazaacenapthylene. It has completed phase III clinical trials for the treatment of uncomplicated urogenital gonorrhea with positive outcomes and was approved by the FDA for the treatment of uncomplicated urinary tract infections.^3-5^

**Figure:**
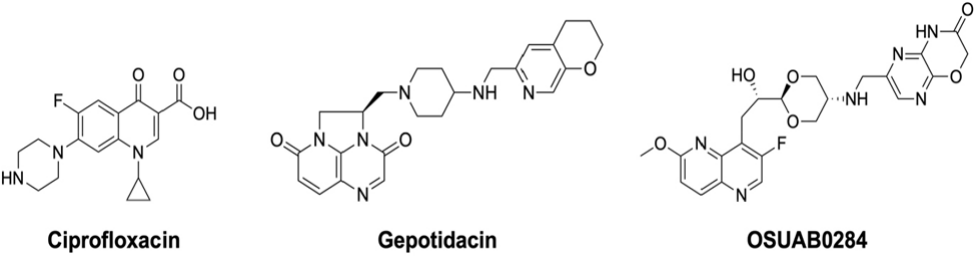
Antimicrobial Agents The compounds analyzed in this work: ciprofloxacin (FQ), gepotidacin (triazaacenaphthylene), OSUAB0284 (NBTI)

**Methods:**

Beyond its clinical success, gepotidacin displayed potent activity against wild-type and FQ-resistant strains of *B. anthracis in vitro* and *in vivo*.^6^ Consequently, we analyzed the effects of OSUAB0284 in parallel to ciprofloxacin (an FQ) and gepotidacin in cell- and enzyme-based *B. anthracis* experiments.

**Results:**

Ciprofloxacin, gepotidacin, and OSUAB0284 displayed potent antibacterial activity against cultured *B. anthracis* (MIC_90_ = 0.06 µg/mL, 0.5 µg/mL, and 0.25 µg/mL, respectively). OSUAB0284 and gepotidacin induced high levels of single-stranded DNA cleavage mediated by purified wild-type *B. anthracis* gyrase and topoisomerase IV, a mechanism distinct from FQs. DNA cleavage activity was maintained against FQ-resistant gyrase enzymes (GyrA^S85L^ and GyrA^E89K^). OSUAB0284 and gepotidacin induced even higher levels of DNA cleavage with FQ-resistant topoisomerase IV enzymes (ParC^S81Y^ and ParC^E85K^) compared to wild-type. OSUAB0284 and gepotidacin inhibited the catalytic function of wild-type *B. anthracis* gyrase and topoisomerase IV and maintained activity with the FQ-resistant enzymes.

**Conclusion:**

An inhalation anthrax model in mice treated with OSUAB0284 is ongoing. Our results suggest that NBTIs are potential therapeutic candidates for use against *B. anthracis*.

**Disclosures:**

Mark Mitton-Fry, PhD, Pfizer: Stocks/Bonds (Public Company)|Viatris: Stocks/Bonds (Public Company)

